# Reversing Effect of Insulin on Local Anesthetics-Induced Sciatic Nerve Block in Rats

**DOI:** 10.1155/2019/4252349

**Published:** 2019-03-11

**Authors:** Jong Min Kim, Seok Hwa Choi

**Affiliations:** Veterinary Medical Center, College of Veterinary Medicine, Chungbuk National University, Cheongju 361-763, Republic of Korea

## Abstract

**Background:**

Local anesthetics are used in various purposes from topical and infiltration anesthesia to peripheral nerve or central neural blockade. Even though local anesthetics are relatively safe, they can have some toxic and adverse effects. Prolonged sensory and motor block is another example of an unwanted complication. The primary objective of this study was to determine whether insulin has a reversal effect on the peripheral (sciatic) nerve block with lidocaine or bupivacaine.

**Methods:**

The surgically exposed sciatic nerves in rats were blocked with lidocaine or bupivacaine, and then 0.1 ml of normal saline or 0.1 ml normal saline containing 0.1 IU a short-acting form of insulin was administrated per body in each group. Before and after sciatic nerve block, as well as until recovery from the nerve block after normal saline or insulin treatment, nerve conduction studies such as monitoring loss and recovery of the waveforms and amplitudes were performed to evaluate the status of motor nerve conduction.

**Results:**

Complete recovery time of nerve conduction status in lidocaine + normal saline group was 58 ± 16 min, whereas that in lidocaine + insulin group was 17 ± 3 min and the difference was statistically significant (*p *< 0.01). Complete recovery time of nerve conduction status in bupivacaine + normal saline group was 116 ± 16 min and that in bupivacaine + insulin group was 36 ± 4 min and the two groups were significantly different (*p *< 0.01).

**Conclusions:**

Insulin can reverse peripheral nerve block induced by lidocaine or bupivacaine.

## 1. Introduction

Local anesthetics are widely used in various fields, but prolonged effects of local anesthesia can result in undesired condition. This is frequently associated with diminished ability to perform normal activities such as speaking, eating, drinking, and smiling after dentistry treatment. Accidental biting of the lips or tongue is of particular concern in children [1]. To prevent those complications, phentolamine mesylate (*α* adrenergic antagonist: vasodilation) was used to shorten the duration of local anesthesia in case of local anesthetics in combination with epinephrine (*α* adrenergic agonist: vasocontraction) [1]. Phentolamine has been shown to enhance systemic absorption of local anesthetics and thus normal lip sensation recovered at 70 min in phentolamine group compared to 155 min in the placebo group [1]. However, local anesthetics that do not contain epinephrine cannot be reversed in this manner. As another example, epidural infusion can resolve local anesthesia within two hours, but this method occasionally results in prolonged postoperative motor blockade, contributing to patient anxiety and extended stay in the recovery room [2]. Intermittent epidural injections of a crystalloid solution during bupivacaine-induced epidural anesthesia have been reported to shorten the duration of bupivacaine-induced motor blockade without compromising sensory analgesia [2]. Finding the cause and appropriate treatment for persistent nerve blockade is needed, and drugs and/or therapies which can be used for reversing nerve blockade within a reasonable time frame may hold promising clinical values.

Local anesthetics prevent rapid influx of Na^+^ into the nerve axons, and this in turn produces the action potential in the nerve [3].* In vitro* studies have shown that local anesthetics inhibited the transient outward K^+^ current and repolarization in rat neocortical neurons [4]. Also, Ca^2+^ current was blocked by local anesthetics in rat dorsal root ganglia neurons [5]. In contrast, insulin has been shown to enhance the transient outward K^+^ current and repolarization [6] Ca^2+^ current in the supraoptic nucleus neurons in rat was shown to be increased by insulin [7]. This finding led us to hypothesize that the insulin might reverse local anesthetics-induced peripheral nerve block and thus can be a physiological antagonist. To prove this hypothesis, this study was conducted to determine whether insulin has a reversal effect on the peripheral (sciatic) nerve block with lidocaine or bupivacaine.

## 2. Materials and Methods

### 2.1. Overview

All experimental agents, including local anesthetics and insulin, were commercially available drugs: 2% lidocaine (Daehan Pharm., Korea), 0.5% bupivacaine (Hana Pharm., Korea), and a short-acting insulin, Humulin R (Eli Lilly, USA).

In the previous study, Cho et al. reported that insulin reversed bupivacaine-induced cardiac depression in dogs [8]. They described that bupivacaine-induced cardiac depression by blocking transient outward K+ and Ca2+ currents of ventricular myocyte. They showed that insulin stimulated outward K+ and Ca2+ currents of ventricular myocyte and this phenomenon would lead to reverse the anesthetic effect of bupivacaine. Based on this concept, we hypothesized that insulin could reverse local anesthetic-induced peripheral nerve block in this study. They used insulin (1 IU/kg) intravenously, but we tried to titrate insulin dose (0.4, 4, and 40 IU/kg) and found that 0.4 IU/kg of insulin was enough to reverse local anesthetics-induced nerve block in the rat in the pilot study (data not shown).

After lidocaine- or bupivacaine-induced sciatic nerve block in rats, 0.1 ml of normal saline (lidocaine + normal saline group), 0.1 ml normal saline containing 0.1 IU of dose insulin (lidocaine + insulin group), 0.1 ml of normal saline (bupivacaine + normal saline group), or 0.1 ml normal saline containing 0.1 IU of dose insulin (bupivacaine + insulin group) were administered. Eight independent Sprague Dawley rats (total 32 rats, mean weight; 250 g) were tested in each group.

This study was approved by the Institutional Animal Care and Use Committee of Chungbuk National University (IACUC approval number; CBNU-198-1002-01).

### 2.2. Surgical Procedure and Evaluation of Nerve Conduction Status

Zoletil® 20 mg/kg and xylazine 5 mg/kg were injected intraperitoneally for general anesthesia. After surgical preparation, all the left sciatic nerves were carefully exposed under a surgical microscope. All fat tissues around the nerve were removed.

**Figure 1:**
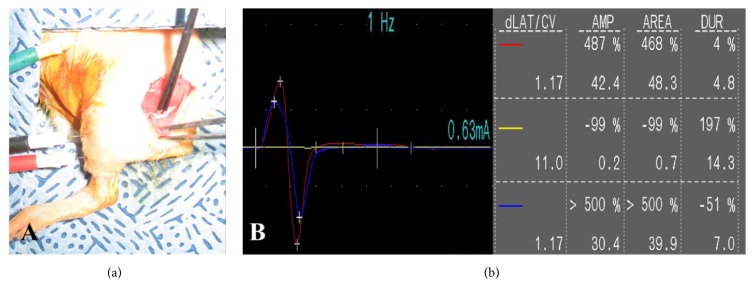
Evaluation of motor nerve conduction in the sciatic nerve in the rat. (a) The sciatic nerve is stimulated with a bipolar intraoperative stimulation device. Recording needles are placed in the cranial tibial muscle belly. (b) Serial recordings of motor nerve conduction in prelocal anesthetic application (red line), after sciatic nerve block by local anesthetic application (yellow line), and after recovery of local anesthetic nerve block (blue line).

Nerve conduction of the sciatic nerve was measured using a Keypoint^TM^ Portable (ALPINE Biomed., Denmark) electromyography device. Sciatic nerve was stimulated with a single electrical pulse (0.1 ms duration, 0.6-0.63 mA intensity). The compound muscle action potentials of the cranial tibial muscle were recorded by means of monopolar needles (28 G) inserted in the muscle belly (Figure 1(a)).

Normal amplitude of sciatic nerve was measured in each rat (Figure 1(b); red line). For lidocaine + normal saline or bupivacaine + normal saline groups, 0.1 mL of 2% lidocaine or 0.5% bupivacaine was instilled into the exposed sciatic nerve. The onset time of local anesthesia was measured by nerve stimulation every minute. When the motor nerve block was confirmed (Figure 1(b); yellow line), the remaining drug was wiped out from the nerve area using a gauze and then 0.1 mL of normal saline was instilled. Until recovery from local anesthesia, nerve stimulation was performed every 2 min. When small amplitude of the action potential appeared, the recovery from local anesthetic was considered initiated, and then the “start” time was measured. It was defined as “complete” when the amplitude of the action potential amplitude recovered as much as preanesthesia level (Figure 1(b); blue line).

For lidocaine + insulin and bupivacaine + insulin groups, when lidocaine- or bupivacaine-local anesthesia was induced, the remaining drug liquid was removed using a gauze, and 0.1 mL of normal saline containing 0.1 IU of a short-acting insulin was instilled. Until recovery from local anesthesia, nerve stimulation was tested every 2 min until start and complete action potential appeared and those times were measured.

After recovery from local anesthesia, general suture of the fascia lata and skin was performed. After recovery from general anesthesia, cefazolin 50 mg/kg and tramadol 10 mg/kg were injected subcutaneously. Rats were returned to* ad libitum* feeding until 10 hours after insulin treatment and euthanized after blood collection.

### 2.3. Measurements of Potassium and Glucose

For lidocaine + insulin or bupivacaine + insulin groups, the concentrations of K^+^ and glucose which would be affected by insulin administration were measured before and 10 hours after insulin treatment.

### 2.4. Statistical Analysis

All results were expressed as means ± standard deviation (SD) and were analyzed by Student's t-test in software Excel. A *p* value < 0.05 was considered statistically significant.

## 3. Results

### 3.1. Evaluation of Nerve Conduction Status

Onset time (time to disappearing amplitude of the action potential) of lidocaine- or bupivacaine-induced anesthesia onto the sciatic nerve was 1.5 ± 0.7 or 5.4 ± 4.5 min, respectively.

Times from start to complete recovery by local anesthetics-induced nerve blocking were 1-10 min. Recovery from the local anesthetics-induced nerve blocking was faster in both groups of lidocaine + insulin and bupivacaine + insulin than those in individual control groups and this difference was statistically significant (*p* < 0.01; Table 1).

### 3.2. Measurements of Concentration of K^+^ and Glucose

For lidocaine + insulin or bupivacaine + insulin groups, the concentrations of plasma K^+^ before and 10 hours after insulin treatment were 4.7 ± 0.1 mmol/L or 4.5 ± 0.2 mmol/L, and blood glucose levels were 84 ± 5 mg/dL or 79 ± 6 mg/dL, respectively. There were no statistically differences in the levels of K^+^ and glucose before and 10 hours after insulin treatment.

## 4. Discussion

We conducted this study to confirm the effectiveness of short-acting insulin for reversing local anesthetics-induced peripheral nerve block. The results showed that insulin infusion was effective in shortening duration of local anesthetic-induced nerve block in rats. The complete reversal from local anesthetics-induced nerve block was enhanced after the insulin infusion (Lidocaine + insulin; 17 ± 3 min, Bupivacaine + insulin; 36 ± 4 min) as compared with saline-treated control (Lidocaine + normal saline; 58 ± 16 min, Bupivacaine + normal saline; 116 ± 16 min) rats. Local anesthetics inhibit voltage gated-Na^+^ channel, transient outward K^+^ current, and Ca^2+^ current in the peripheral nerves [3–5]. Although it is unknown whether insulin have direct effect on voltage gated-Na^+^ channel which has been already bound with local anesthetics, transient increases in outward K^+^ current and repolarization [6] and in Ca^2+^ current [7] in the presence of insulin may play a role in reversing local anesthetics-induced peripheral nerve block.

We used insulin to reverse local anesthetic-induced sciatic nerve block by above-mentioned mechanism. One interesting report showed that magnesium sulfate significantly diminished the effects of local anesthetics in rat sciatic nerve block [9]. The antagonistic mechanism of magnesium sulfate is unclear, but they speculated that action of magnesium sulfate is independent of the local anesthetics receptor within the Na+ channel. Magnesium sulfate is another option to shorten the effect of local anesthetic-induced nerve block.

In view point of neurobehavioral examination to evaluate nerve block status, one group used proprioception, motor function (extensor postural thrust), and nociceptive reaction to assess nerve block and recovery from local anesthetics every 15 min in rat sciatic nerve [10]. We evaluated status of nerve block or recovery from local anesthetics by measuring nerve conduction in this experiment. The recorded compound muscle action potentials are superior to measure initiation or complete recovery time from local anesthetic nerve block compared to previously mentioned methods (Figure 1(b)).

There were no statistically differences in the levels of K^+^ and glucose before and 10 hours after insulin treatment. This phenomena may be explained by the fact that relatively low dose (about 0.4 IU/kg) of a short-acting insulin in this experiment was used compared to other experiment (about 6-36 IU/kg) [11] of diabetic rat and* ad libitum* feeding after insulin treatment.

Limitation of this study was that the dosing of the local anesthetics was not constant, because the drugs had to be applied to an open wound, exposed nerve and then the residual solution was wiped away after the full block had been developed. Since the block onset time depended on the formulation, different times would pass and different amounts of drug would be absorbed into the local tissues or into the circulation. This would affect the duration of the block. Because the way of treating local anesthetics in this study was not clinically relevant, if more standard delivery method, percutaneous injection of a fixed volume is used, the confounding effect would be avoided.

Another limitation of this study was lack of positive control. Because local anesthetics inhibit voltage gate-Na+ channel, transient outward K+ current, and Ca2+ current, it is conceivable that ion channel agonists for Na+ (e.g., dimethyl lithospermate B) [12], K+ (e.g., Diazoxide; The potassium channel openers that target the ATP-sensitive potassium channels; Opening of KATP channels causing K+ efflux, hyperpolarization of the cell membrane) [13], and Ca2+ (e.g., Bay K 8644) [14] would be served as positive controls (single or combination of each agonist). Further study is needed to compare reversing potency between insulin and upper mentioned positive controls.

In summary, we found that insulin can reverse peripheral local anesthesia induced by lidocaine and bupivacaine. This suggests that insulin can be used in the treatment of undesired prolonged effects of local anesthesia as a physiological antagonist.

## Figures and Tables

**Table 1:** Short-acting insulin can reverse lidocaine- or bupivacaine-induced sciatic nerves local anesthesia.

	Sciatic nerve recovery (min)	
	start	complete
Lidocaine + normal saline	48 ± 6	58 ± 16
Lidocaine + insulin	16 ± 2^#^	17 ± 3^#^
Bupivacaine + normal saline	108 ± 19	116 ± 16
Bupivacaine + insulin	30 ± 5^*∗*^	36 ± 4^*∗*^

Results are presented as mean ± SD (n = 8).

“Start” means that small amplitude of the action potential appears as recovery from local anesthesia. “Complete” means that when the action potential amplitude is recovered as much as preanesthetics level.

Insulin is a short-acting insulin, Humulin R®.

^#^Significant difference from lidocaine + normal saline group at *p* < 0.01.

^*∗*^Significant difference from bupivacaine + normal saline group at *p* < 0.01.

## Data Availability

The data used to support the findings of this study are included within the article.
